# Spatio-Temporal Characteristics of Landscape Ecological Risks in the Ecological Functional Zone of the Upper Yellow River, China

**DOI:** 10.3390/ijerph182412943

**Published:** 2021-12-08

**Authors:** Fuwei Qiao, Yongping Bai, Lixia Xie, Xuedi Yang, Shuaishuai Sun

**Affiliations:** 1 College of Economics, Northwest Normal University, Lanzhou 730070, China; qfw279@nwnu.edu.cn; 2 College of Geography and Environmental Science, Northwest Normal University, Lanzhou 730070, China; baiyp@nwnu.edu.cn; 3 College of Earth and Environmental Sciences, Lanzhou University, Lanzhou 730070, China; yxdlz01@163.com; 4 Northwest Branch of Shanghai Tongji Urban Planning & Design Institute Co., Xi’an 710000, China; sun62817@126.com

**Keywords:** land use/land cover change, landscape pattern index, landscape ecological risk, EFZUYR

## Abstract

The Ecological Functional Zone of the Upper Yellow River (EFZUYR) is a critical water-catching area in the Yellow River Basin, the ecological security of which affects the sound development of the ecosystem in the entire basin. Recently, significant land use changes have aggravated regional ecological risks and seriously affected the sustainable development of EFZUYR. In this context, this paper provides an in-depth study of the ecological risks caused by land use landscape changes. With the help of land use data and dynamic degree analysis, the land use transfer matrix, and the landscape pattern index, this paper quantifies the distribution trends of land use landscape patterns in EFZUYR from 1990 to 2018. In addition, this research explores the temporal and spatial dynamic distribution characteristics of landscape ecological risks in this functional zone. The research results show the following: (1) The transfer of land use in EFZUYR from 1990 to 2018 mainly occurred among cultivated land, grassland, and woodland, with the transferred area accounting for 87.16% of the total changed area. (2) The fragmentation degree of built-up areas is 0.1097, 0.1053, 0.0811 and 0.0762 in 1990, 2000, 2010 and 2018, respectively, with a decreasing trend. The dominance degree of grassland has been maintained at the highest level for a long time, with all values above 0.59. The separation degree and the interference degree of built-up areas were the highest and the values of the four periods were above 1.2 and 0.44, respectively. The loss degree of water was the highest, with a value above 0.67, while the value of other land use was mostly below 0.4. (3) The landscape ecological risk of EFZUYR presented a fluctuating rising, falling, and then rising trend. The spatial distribution characteristic of EFZUYR presented “high in the north and south, low in the middle.”, which has been maintained for a long time. The proportion of low-risk areas is as high as 70%, and the overall ecological risk of the region was low. However, the ecological risk of some areas, such as Linxia City and Magu County, increased. These findings can provide theoretical support for land use planning and achieving sustainable development of EFZUYR.

## 1. Introduction

Watershed is an important area in which humans engage in social production, and the population distributed in significant watersheds in the world is as high as 2.24 billion, accounting for about one-third of the world’s population [1]. As the fifth-longest river in the world and the second-longest river in China, the Yellow River is famous for being among the birthplaces of the ancient Chinese civilization and the most extensive sand content. In 2019, China identified the ecological protection and high-quality development of the Yellow River Basin as the fifth national strategy. The policy of ecological environment management has reached an unprecedented high, and the construction of the ecological security pattern of the Yellow River Basin has continued to advance [2]. With these efforts, breakthroughs have been made in restoring the ecosystem and the environmental protection of the Yellow River Basin. However, due to the fragility of the ecological environment of the Yellow River Basin and the intensification of human interference, the ecological security problems of the entire basin have taken severe forms (such as soil erosion, land degradation, and weakening of ecosystem function). Ecological governance remains highly arduous [3]. Since 2001, to promote the implementation of ecological security protection project in the Yellow River Basin, the relevant departments of the State of China began to study the zoning of ecological functions and clarify the crucial areas for safeguarding national ecological security. The ecological functional zone is a comprehensive ecosystem that integrates conserving river sources, mediating the relationship between humans and nature, and promoting ecological protection. It plays a critical role in maintaining the safety of the region’s ecological environment and the whole country. The protection of the stable development of the ecological functional zone depends on the rational use of land [4]. Located in the north-eastern part of the Tibetan Plateau, EFZUYR is known as the reservoir of the Yellow River and has a prominent ecological strategic position. Land use or land cover change (LUCC) in China has undergone a complex series of changes over the past three decades due to fast economic growth and the adoption of several land use policies [5], and EFUYR is no exception. However, assessing the ecological risk of EFZUYR based on LUCC is particularly important for ecological restoration and water conservation.

Scientific regional ecological risk assessment can provide an essential basis for the policy formulation, planning, and land management of natural resource sustainability [6]. The demand for environmental decision-making and planning management has promoted the continuous expansion of the scope and content of ecological risk research and, as a result, ecological risk assessment has received increasing attention from academic circles [7]. Moreover, ecological risk assessment has changed from a traditional ecological risk assessment to a regional ecological risk assessment and a landscape ecological risk assessment [8]. Compared with the traditional ecological risk assessment, the landscape ecological risk assessment can better express spatial heterogeneity, which has become the most popular method for assessing ecological risk [9]. Numerous scholars have made breakthroughs in landscape ecological risk. The typical research areas selection mainly covers research areas such as watershed [10], cities [11], mountain [12,13], wetland [14] and nature reserves [15,16]. The research content mainly focuses on the assessment of landscape ecological risk [17,18], spatial and temporal patterns [16], and the impact of different factors on landscape ecological risk [19]. With regard to research methods, the measurement and calculation of landscape ecological risk mainly include the risk “source-collection” method and landscape index method [11]. The evaluation method based on risk source collection is more suitable for evaluating specific ecological risks with apparent stress factors in certain areas. However, this method must be combined with the specific ecological processes or disaster risks to identify the landscape type that promotes or hinders the sound development of the ecosystem [20] and does not take landscape heterogeneity and ecosystem change patterns into consideration. However, the evaluation method based on the landscape index method focuses on assessing ecological risk from the spatial pattern of the landscape, which can comprehensively evaluate the ecological impact and cumulative effects of multiple risk sources in the landscape mosaic [21]. In this method, land use/cover change (LUCC) is the basis of ecological risk assessment [22]. Compared with the evaluation method of risk source collection, the landscape index method quantitatively evaluates the overall ecological quality of the region and focuses on analysing the spatial-temporal variation characteristics of risks and the risks of land use status to ecological functions and processes. This is the reason that, in recent years, landscape ecological risk assessment based on the landscape index method has witnessed its most comprehensive application.

EFZUYR is an important ecological barrier of the Yellow River, with important functions of water recharge, maintaining biodiversity and regulating regional climate, and has an irreplaceable role in maintaining the water resources and ecological security of the Yellow River basin. Therefore, this study quantitatively analyzed the spatial distribution characteristics of EFZUYR ecological risks using the land transfer matrix, landscape ecological risk model and cold-hot spot analysis, which fills the knowledge gap of landscape ecological risk assessment in the upper Yellow River Basin. The main goal of this study was to assess the landscape ecological risk of EFZUYR based on the changes in LUCC using the landscape index approach. The specific objectives of this study were (1) to analyze the area change of EFZUYR land use and the transfer characteristics between different land uses from 1900 to 2018, (2) to explore the change characteristics of the landscape index of EFZUYR land use, and (3) to evaluate the spatial and temporal evolution characteristics of EFZUYR landscape ecological risk.

## 2. Materials and Methods

### 2.1. Study Area

**Figure 1:**
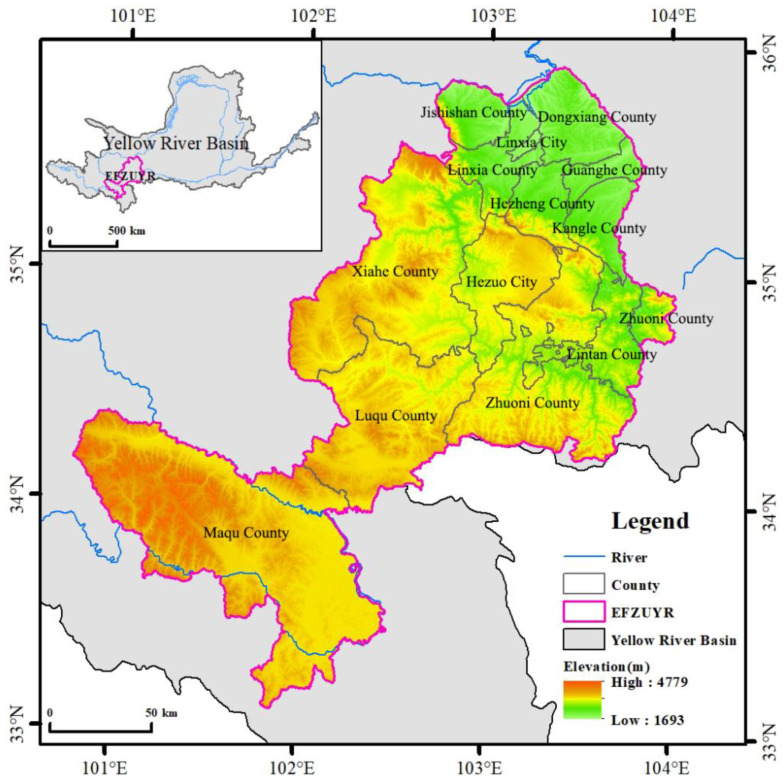
Overview of the study area in the background of Yellow River Basin.

EFZUYR is located in the north-eastern extension of the Qinghai-Tibet Plateau and is the drainage divide between the Yellow River Basin and the Yangtze River Basin in China. Its unique geographical location and natural geographic characteristics have determined its fundamental ecological attributes, such as an ecological transition zone and a fragile zone, which is of great significance for maintaining social stability and ecological security [23]. The rapid socio-economic development and overuse of land have led to an increase in regional ecological risks. In addition, EFZUYR is not only the largest plateau wetland at the eastern end of the Qinghai-Tibet Plateau and a vital water replenishment region for the upper reaches of the Yellow River, but also an important conservation area for rare flora and fauna of the Tibetan Plateau. Its administrative region includes most counties in Linxia Hui Autonomous Prefecture (Linxia Prefecture) and Gannan Tibetan Autonomous Prefecture (Gannan Prefecture) in Gansu Province of China (Figure 1). The terrain of the study area is high in the southwest and low in the northeast, with altitudes ranging from 1500 to 4900m. The vegetation is mainly grassland, wetland, and mountain woodland, such as alpine meadow, which is mainly composed of Carex and Kobresia, and subalpine shrub, which is mainly composed of Rh. przewalskii and Rh. rufum. The climate is temperate continental monsoon with a large diurnal temperature difference. The annual average temperature in the southwest area is 4 °C. Additionally, precipitation in the southwest is unevenly distributed with large interannual variations. Linxia Prefecture and Gannan Prefecture are the gathering and living areas for Hui, Tibetan, Dongxiang, Salar, and Tu ethnic groups. Data from the seventh census of China shows that the resident population of the study area is 2,143,900.

### 2.2. Data

**Figure 2:**
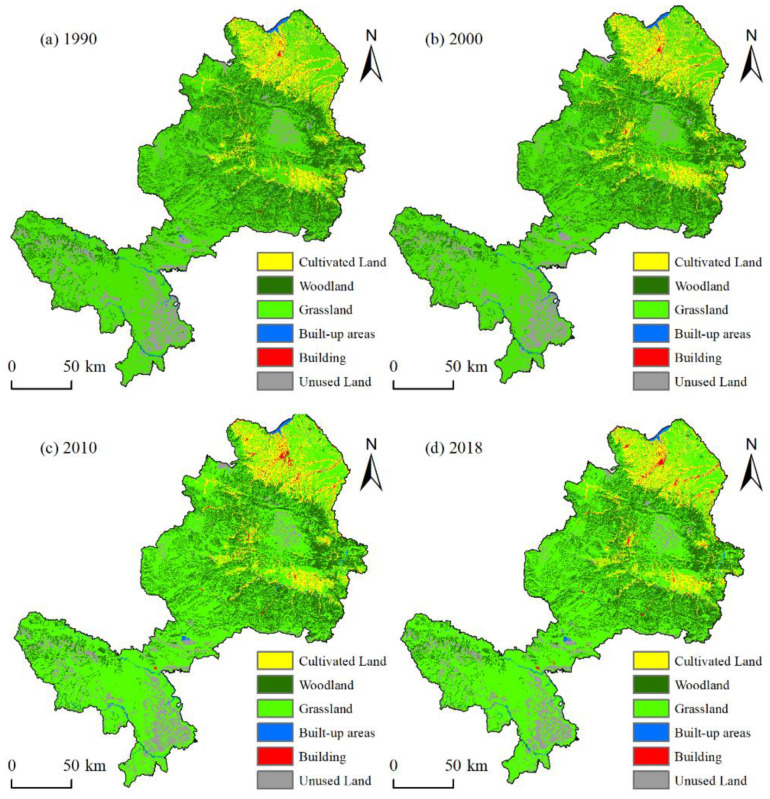
Land use of EFZUYR from 1990 to 2018. (**a**) 1990 (**b**) 2000, (**c**) 2010 and (**d**) 2018.

The land use data used in this paper are from 1990, 2000, 2010 and 2018, which were obtained from the Resources and Environmental Science and Data Center (http://www.resdc.cn/; accessed on 5 March 2021) and have a spatial resolution of 30 m × 30 m. These data have been widely used and their accuracy meets the criteria of the present study. Referring to the classification standard of China’s land use status (GB/T21010—2007) and considering the characteristics of different land use in EFZUYR, this study utilized the reclassification function of ArcGIS 10.6 to classify the land use data into six categories: cultivated land, woodland, grassland, water, built-up areas and unused land (Figure 2). The Digital Elevation Model (DEM) data comes from the Geospatial Data Cloud (http://www.gscloud.cn/; accessed on 5 March 2021), with a spatial resolution of 90 m× 90 m.

### 2.3. Methods

#### 2.3.1. Land Use Dynamic Degree

The dynamic degree of land use is mainly used to describe the area change in land use in a certain period, to express the intensity of land use in a region and the differences among different land uses, periods, or regions. In this study, the dynamic degree of various land uses of EFZUYR for 1990–2000, 2000–2010, 2010–2018 and 1990–2018 was calculated to analyze the land use changes in the study area. The formula for the dynamic degree of land use is detailed in the literature (Equation (A1)) [24].

#### 2.3.2. Landscape Ecological Risk Index

Landscape ecological risk refers to the possible adverse consequences of the interaction between the landscape pattern and ecological process under the influence of natural or human factors [20]. The landscape ecological risk index (ERI) consists of the landscape disturbance index and the landscape vulnerability index, reflecting the relationship between landscape patterns of land use and ecological risk [25]. Based on fully considering the impact of land use and landscape variability on the ecological environment under human activity disturbance, this study used the landscape pattern index to establish the assessment method of the landscape ecological risk of EFZUYR. The formula for ERI is detailed in the literature (Equation (A2)) [16,26].

Combining the research results of Chen [26] and the actual condition of the research area, this study divided the ecological risk of the ecological function area in the upper Yellow River Basin into five grades with an equal interval division method: the lowest risk area (ERI ≤ 0.20), the lower risk area (0.20 < ERI ≤ 0.22), the medium risk area (0.22 < ERI ≤ 0.24), the higher risk area (0.24 < ERI ≤ 0.26), and the highest risk area (ERI > 0.26).

**Figure 3:**
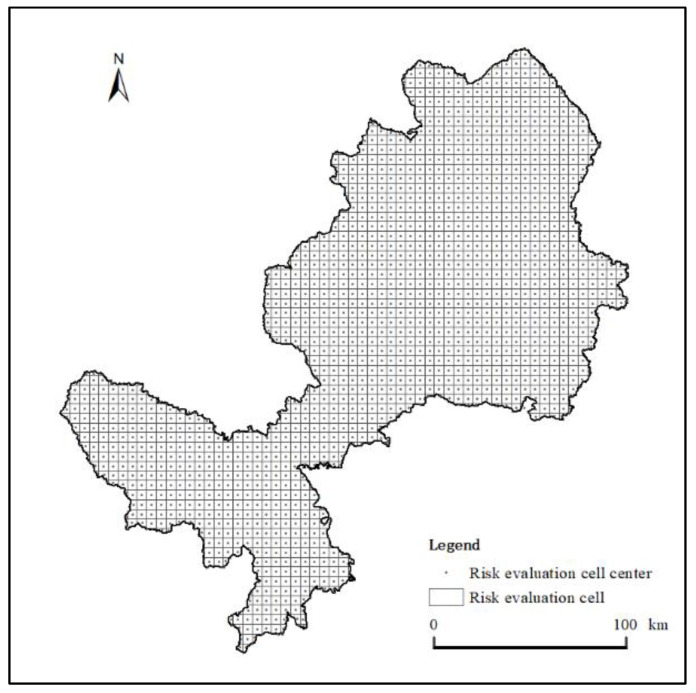
Grid division of EFZUYR ecological risk.

To present the EFZUYR landscape ecological risk index’s spatial distribution characteristics, this study utilized landscape ecology theory to conduct equidistant sampling of land use data in 1990, 2000, 2010 and 2018. After many experiments and comparisons, it was found that 5 km × 5 km is the optimal scale for ecological risk research in the area, so the research area was divided into 5 km× 5 km grids (Figure 3). In data processing, grids were used as small research units for spatial sampling. Ecological risk values were calculated for all grids, and landscape ecological risk values were assigned to the center of each ecological risk unit.

#### 2.3.3. Cold-Hot Spot Analysis

Cold-hot spot analysis is often used to find the spatial distribution characteristics of landscape ecological risks in the study area. In this study, the Getis-Ord General G was used to explore the overall pattern and trend of landscape ecological risk in the study area, and the Getis-Ord Gi* index of landscape ecological risk was used to describe the spatial distribution of cold and hot spots of ecological units. Hot spots indicate areas where high values of landscape ecological risk are clustered, and cold spots represent areas where low values of landscape ecological risk are clustered. The formulas for the Getis-Ord General G and the Getis-Ord Gi* are detailed in the literature (Equations (A3)–(A5)) [27].

## 3. Results

### 3.1. Change Characteristics of Land Use

#### 3.1.1. Area Changes in Land Use

This study performed a statistical analysis on the land use data of EFZUYR of four periods from 1990 to 2018 and obtained statistics on the area changes in the six land use types in functional areas (Table A1). During the research period, grassland and woodland were the main land use of EFZUYR, and the characteristics of the number changes in each land use type were widely different. In 1990, the ranking of the EFZUYR land use area ratio was as follows: grassland (61.07%) > woodland (21.82%) > cultivated land (8.72%) > unused land (6.67%) > water (0.92%) > built-up areas (0.72%); by 2018, the proportion transitioned into the following order: grassland (60.81%) > woodland (21.68%) > cultivated land (8.87%) > unused land (6.57%) > built-up areas (1.14%) > water (0.93%)). It can be seen that the sum of EFZUYR grassland and woodland maintained at more than 80%, and the increased speed of built-up areas exceeded water. Considering the number change degree of each land use type, unused land had the most significant reduction in area. From 1990 to 2018, unused land witnessed a reduction of 72.13 km² and a reduced rate of 3.03%, and the grassland area showed a fluctuant decreasing trend in area, with a decrease of 130.13 km². However, due to the large base of grassland area, the reduction area only accounted for 0.61% of the total area, and thus the change rate was rather low; woodland also showed a fluctuant decreasing trend in area, with a decrease of 64.94 km² compared with 1990 and a reduced rate of 0.85%. In EFZUYR, the land use with the largest increased area was built-up areas which increased by 148 km² during the research period, with an increased rate of 59.06%. From 1900 to 2018, the cultivated land area showed a fluctuating increasing trend of 47.07 km^2^, and the rate of increase was 1.54%. Water also showed a trend of fluctuant increase in area and increased by 4.57 km², with an increased rate of 1.42%. The above statistics show that woodland and grassland cover a greater portion of ecological land use in EFZUYR, and the built-up areas showed a rapid increasing trend.

#### 3.1.2. Dynamic Changes in Land Use

According to the dynamic degree of EFZUYR land use (Table 1), differences in the change rate of land use in different periods can be noticed. Cultivated land showed an increasing trend first and then decreased slowly from 1990 to 2018. The overall change showed the characteristics of growth, with a change rate of 0.04 %. The increase was the most obvious from 1990 to 2000, with a growth rate of 0.74 %. Woodland showed a decreasing trend first and then increased from 1990 to 2018. The overall change showed a decreasing characteristic, with a reduction rate of 0.02%. From 1990 to 2000, the rate of decrease was the largest, with a reduction rate of 0.16%. Grassland first showed a decreasing trend, then increased and decreased from 1990 to 2018. The overall change showed a characteristic of decreasing, with a reduction rate of 0.02%. The reduction rate in 1990–2000 and 2010–2018 was the same-both 0.06%. Water first showed a decreasing trend and then increased from 1990 to 2018. The overall change showed an increasing characteristic, with a growth rate of 0.04%. It increased rapidly from 2010 to 2018, with a growth rate of 6.21%. Built-up areas showed a continuous increasing trend from 1990 to 2018, with a growth rate of 1.51%, and was the fastest growing from 2000 to 2010, with a growth rate of 3.53%. Unused land showed a constant trend first and then decreased, and the overall change showed the decreasing characteristics. The decrease in 2000–2018 was the largest, with a decrease rate of 0.29%.

**Figure 4:**
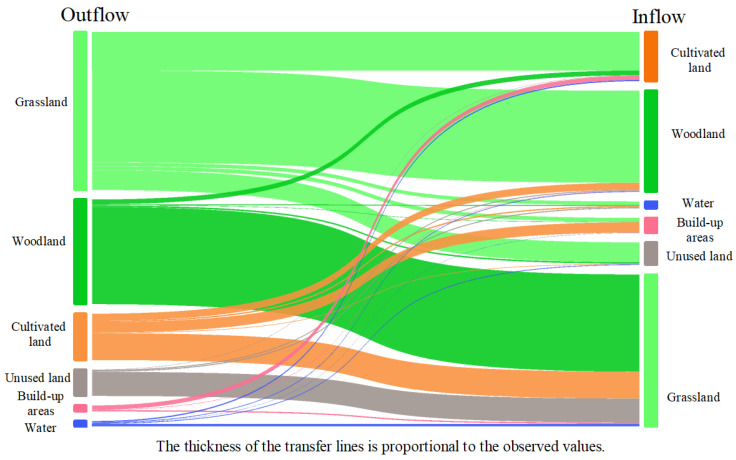
Transformation distribution of different land use from 1990 to 2018.

This study used the land use transfer matrix to reveal the detailed transfer status among each land use type (Figure 4, Table 2). According to the land use transfer of EFZUYR from 1990 to 2018, the total land use change was 5774.01 km², with a change rate of 16.45%. In the study area, grassland is 2530.28 km², accounting for 7.21% of the total transferred area, which is the largest land use transfer area. These areas were mainly transformed into woodland, cultivated land and unused land. Cultivated land was one of the major land-uses that transferred into other types, mainly grassland, woodland, and built-up areas. The transferring area of cultivated land was 818.61 km², accounting for 2.33% of the transferred land. Built-up areas were the main type of inflow, which was mainly derived from cultivated land and grassland. The area change reached 250.22 km², accounting for 76.80% of the total change area of built-up areas. Woodland mainly transferred into grassland and cultivated land, with a changing area of 1683.49 km², accounting for 22.14% of the total woodland area. Water was mainly transferred from grassland and cultivated land, with a changing area of 115.71 km², accounting for 35.52% of the total water area. Unused land mainly transferred into grassland and woodland, with the transferring area accounting for 14.28% and 1.07% of the total unused land. Overall, compared with built-up areas and cultivated land, the transfer area of other land use was smaller.

In sum, the transfer of cultivated land, grassland, and woodland in EFZUYR was obvious, with a transfer area of 5023.37 km² from 1990 to 2018. The data showed that the expansion of urban built-up areas has taken up many cultivated land resources.

### 3.2. Analysis of Landscape Index Changes of Land Use

The landscape indexes of different land types in 1990, 2000, 2010, and 2018, including the fragmentation degree (C), the separation degree (N), the dominance degree (K), the interference degree (S) and the loss degree (R), were evaluated in this study. From Table 3, the following results can be drawn: (1) There was a relatively small change in the overall fragmentation degree of each land use type. The fragmentation degree of cultivated land, water area, and unused land increased during the research period. The fragmentation degree of woodland and grassland was unchanged. The fragmentation degree of built-up areas showed a decreasing trend, which indicates that built-up areas possess an obvious contiguous development trend, reducing their fragmentation degree. (2) The separation degree index of cultivated land, grassland, and unused land increased, while the index of woodland, grassland, and built-up areas are downward. Therefore, the separation degree of built-up areas was above 1.2, which is much higher than that of other land use type. (3) Grassland had the highest dominance degree, followed by woodland. The unique geographical environment and climatic conditions allowed the alpine grassland and forest ecosystem in EFZUYR to develop into a complete ecosystem, with the extensive distribution of grassland and woodland. (4) Built-up areas had the highest interference degree because people have the greatest interference degree regarding the environment. In addition, the interference degree of cultivated land and unused land showed a continuous increase during the research period. (5) The loss degree was affected by the interference degree and the vulnerability degree, and its development trend is the same as the interference degree. Water area had the highest loss degree among all land types, with the loss degree index value of the four phases exceeding 0.67. Built-up areas had the second-largest loss degree, with the loss degree index value exceeding 0.44. Due to its relatively large vulnerability degree index, water had the most extensive loss degree. Although the vulnerability of built-up areas was relatively low, the degree of loss was also influenced by the degree of disturbance. Therefore, the maximum disturbance degree of built-up areas led to an increased loss degree.

### 3.3. Temporal and Spatial Evolution Characteristics of Landscape Ecological Risk

#### 3.3.1. Temporal Variations of Landscape Ecological Risk

**Figure 5:**
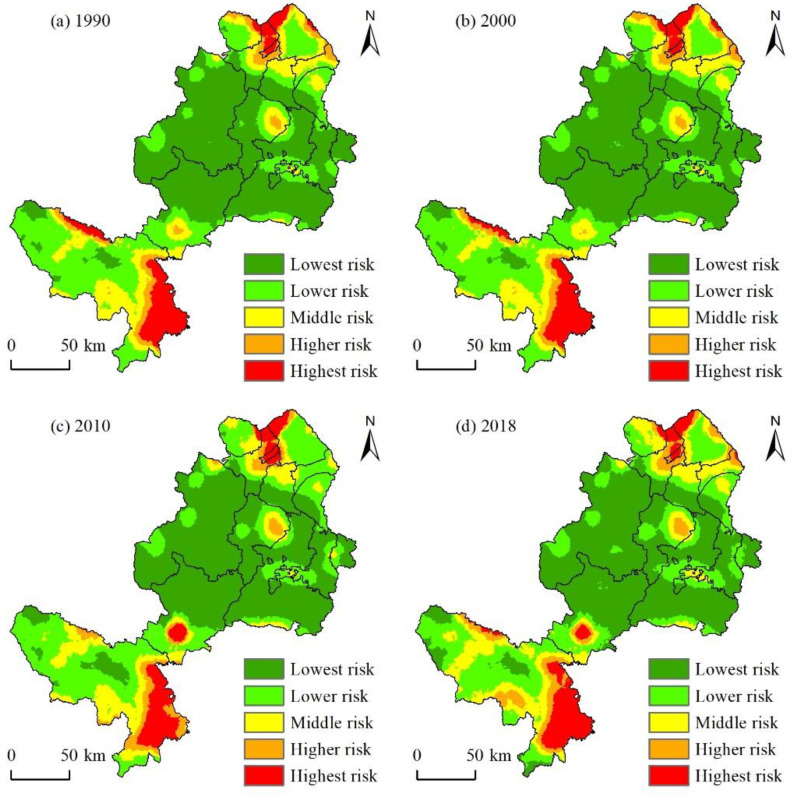
Spatial distribution map of landscape ecological risk. (**a**) 1990; (**b**) 2000; (**c**) 2010; (**d**) 2018.

To clearly express the temporal and spatial characteristics of ecological risks, this study calculated the EFZUYR ecological risk grade area and proportion (Table 4). We visualized ecological risks through the ordinary Kriging interpolation method (Figure 5).

From the characteristic of temporal variations evolution (Table 4), the change in landscape ecological risk in EFZUYR was characterized by an “N” type, with relatively minor changes. Therefore, the ecological risk was relatively stable. As the proportion of low ecological risk areas, including the lowest and lower ecological risk areas, remained above 70% for a long time, the whole area was in a low ecological risk state. Specifically, the average ecological risk values for the four periods of EFZUYR were 0.2122, 0.2126, 0.2115, and 0.2123, showing a fluctuant rising, falling, and then rising trend. However, the change rate was minimal. From the area change in different land use types, the proportion of low ecological risk type shows a decreasing trend from 1990 to 2018, and the area decreased by 1095.75 km², with a decreasing percentage of 3.11%. Among them, the area of the lowest ecological risk regions shows a continuous decline, with a total decrease of 324.00 km². The area of the lower ecological risk regions showed a trend of rising and then falling, with a decrease of 771.75 km². Contrary to the change characteristic in the low-risk regions, the area changes in the medium risk regions showed an “increasing-decreasing- increasing” trend, with an overall increase of 539.25 km². The highest risk regions showed an “increasing-decreasing-increasing” trend in the entire research area, with the total area increasing from 3772.50 km² in 1990 to 4329.00 km² in 2018. Among them, the higher risk regions had a relatively sizeable increasing range, with an increased area of 504.75 km² which accounted for 90.84% of the area change in the high ecological risk regions. The area changes in the highest risk regions increased, but the increased area was only 51.75 km², with an increasing percentage of only 0.15% compared with the increased area in 1990.

#### 3.3.2. Spatial Evolution of Landscape Ecological Risk

From the spatial distribution map of landscape ecological risk (Figure 5), we found that the characteristics of the spatial evolution of EFAUYR ecological risk were that the overall landscape ecological risk was relatively low. The ecological risk level increased in some areas, maintaining a long-term spatial pattern of “high at the north and south ends and low in the middle” from 1990 to 2018. Specifically, the highest-risk areas were mainly distributed in Linxia City, Linxia County and Dongxiang County in the north, and Maqu County and Luqu County in the south. Among them, Luqu County, which did not present the highest risk regions until 2010, has suffered a significant increase in highest risk regions, which has increased to 184.75 km², since 2010. Regions with higher risk were mainly concentrated in Maqu County in the south. In 2018, the area of the higher risk regions in this area reached 1059.75 km², accounting for 52.49% of the higher ecological risk regions in the entire region, followed by Linxia County and Hezuo City in the north. Medium risk regions were mainly distributed in Maqu County. From 1990 to 2018, the area of the medium risk regions in Maqu County accounted for more than 45% of the total area of the medium-risk regions in the research area, and this number exceeded 50% in 2010. The remaining medium ecological risk regions were mainly distributed in Dongxiang County, Jishishan County, Guanghe County, Luqu County and others. The lower risk regions were mainly distributed in Maqu County and Luqu County. The area of the lower risk regions in Maqu County, accounted for approximately 40% of the total area of the lower risk regions in the research area, and this proportion in Luqu County was approximately 10%. The remaining lower ecological risk regions were distributed in Zhuoni County, Xiahe County and other regions. The lowest ecological risk regions were mainly distributed in Xiahe County, Zhuoni County and Luqu County, which are in the central part of EFAUYR. Among them, the area of the lowest ecological risk regions in Xiahe County accounted for approximately 30–35% of the total area of the lowest risk regions in the research area, and the proportions in Zhuoni County and Luqu County were approximately 25% and 17%. The results showed that from 1990 to 2018, EFZUYR had two high risk agglomeration areas for a long time, which were distributed in the south and north. The low-risk area was mainly concentrated in the central area, and the degree of agglomeration showed a downward trend.

#### 3.3.3. Spatial Clustering Characteristics of Landscape Ecological Risk

The spatial clustering of landscape ecological risks can better identify the spatial clustering characteristics of high and low ecological risk regions. According to the General G Index (Table 5), the Observed General G was greater than Expected General G, which indicated that high-value clusters were more obvious. Moreover, the Observed General G in the four periods has an increasing trend, which indicated that the spatial high-value clustering characteristic of the ecological risk in the research area was continuously increasing.

**Figure 6:**
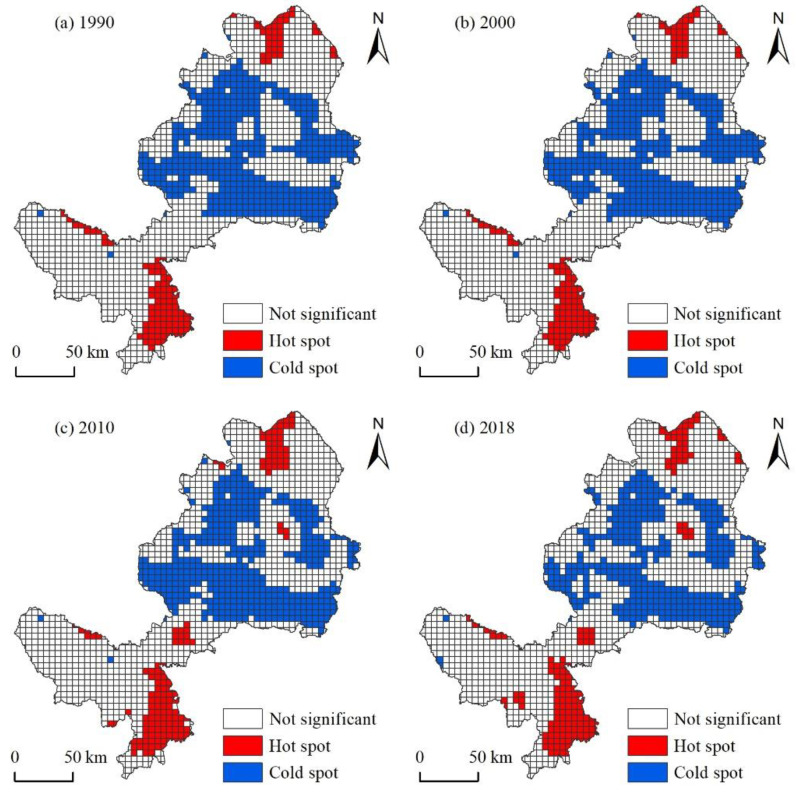
Analysis on Cold and Hot Spots of Landscape Ecological Risks in EFZUYR. (**a**) 1990; (**b**) 2000; (**c**) 2010 and (**d**) 2018.

In terms of spatial distribution characteristics, the spatial clustering pattern of EFAUYR ecological risk has long been characterized by “high in the north and south and low in the central”. In this pattern, the ecological risks of various counties are relatively different. Among them, the ecological risks of Maqu County, Linxia City and Dongxiang County are the highest, and the ecological risks of Lintan County, Zhuoni County and other regions are lower, which forms higher ecological security (Figure 6). Specifically, EFZUYR has long been distributed with two hot spot clustering regions in the north and south. The hot spot regions in the north are mainly concentrated in the Daxia River valley area, and hot spot clustering regions cover the whole area of Linxia City. At the same time, the hot spot regions in the south are mainly concentrated in Maqu County. In addition, the cold spot of the landscape ecological risk is mainly in Zhuoni County and Lintan County, which are in the central part of EFZUYR.

## 4. Discussion

### 4.1. Reasons for Spatial Clustering Characteristics of Landscape Ecological Risks

According to the above research results, EFZUYR has long been distributed with two hot spot clustering regions in the north and south. Although there are two hot spot regions in the north and south of EFZUYR, the causes for the formation are different. The hot-spot regions in the north have a high level of economic development, which means that humans have greater interference with the ecological environment, and the destruction of the ecological environment is severe. In the south, Maqu County has become a hot-spot region. The causes are both natural and anthropogenic. Among them, natural causes are the most fundamental factor in the deterioration of Maqu County’s ecological environment. Anthropogenic causes are an influencing factor but do not play the leading role. Climate warming has led to increased evaporation, surface drought, vegetation degradation, and lake retreat, reducing the stability of the originally fragile ecosystem and weakening its resilience, which has become the main driving force for the degradation of the ecological environment [28]. It is worth noting that the role of anthropogenic causes cannot be ignored [29]. The cold spot of the landscape ecological risk is mainly in Zhuoni County and Lintan County, which are in the central part of EFZUYR. The main reason for this is that there is a large area of forest and grassland in Zhuoni County and Lintan County. The attractive natural ecological environment facilitates the spatial clustering of low values of ecological risks, which constitutes the cold-spot clustering region. Due to temporal and spatial change, the clustering characteristic of the cold-spot region is gradually weakening. In addition, a small hot-spot area in Hezuo City, located in the central part of EFZUYR, was gradually formed and rapidly expanding, mainly due to the fact that Hezuo City is the political, economic and cultural center of Gannan Prefecture. In recent years, with the implementation of the Chinese government’s specific poverty alleviation policy, the level of urbanization in poor areas such as Linxia Prefecture has rapidly increased [30]. At the same time, the continuous improvement of the urban infrastructure, the increasing concentration of the urban population and the increasing proportion of land urbanization means that human interference with the ecological environment is increasing, and ecological risks are rising.

### 4.2. Partition Management of EFZUYR

**Figure 7:**
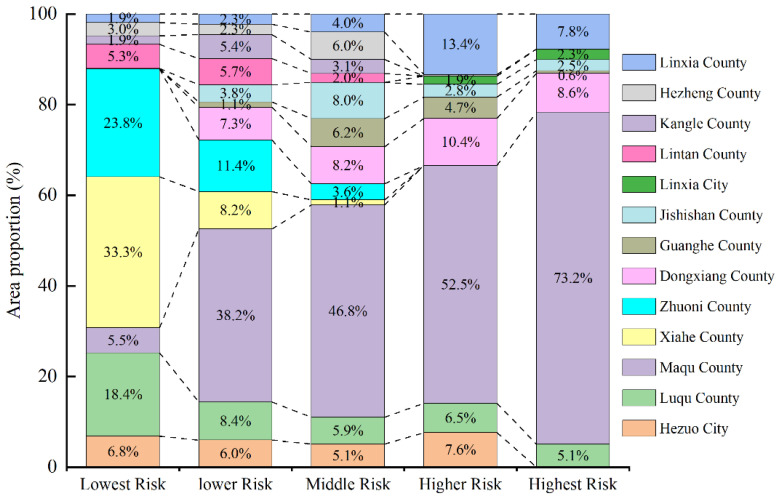
The proportion of the area of the five risk levels in each county (city) in 2018.

The study divided the ecological functional service zones in the upper Yellow River Basin into different levels and calculated the area proportion of the five risk levels in each county (city) in 2018 (Figure 7). It was found that Maqu County, Dongxiang County, and Linxia County are the main distribution areas of the highest, higher, and medium landscape ecological risk areas. The whole area of Linxia City is the highest and higher risk regions, and Xiahe County, Zhuoni County and Luqu County are the main distribution area of the lowest and lower risk regions. The reasons for the spatial difference of ecological risk distribution are very different. First, Maqu County has become a high-risk area mainly due to natural factors. From 1990 to 2018, the annual average temperature in Maqu County showed a significant upward trend. Climate warming led to a reduction in wetlands, grassland degradation, a reduction in biodiversity and the weakening of ecosystem functions [31,32], resulting in a larger proportion of landscape ecological risks in the region. Of course, human economic activities have also exacerbated the ecological risks in the area to a certain extent. Second, the entire area of Linxia City is a high-risk area, and a relatively high proportion of high-risk areas in Linxia Country and Dongxiang County are caused by human activities. Linxia City is the seat of the Linxia Hui Autonomous Prefecture and is the area with the highest degree of economic development in the study area. The population urbanization rate is as high as 88.69%. The land expansion is the most obvious in the study area [30]. This indicates that Linxia City is the most frequent human activity in the study area. In the process of rapid urbanization, the development of the land use landscape has changed greatly, resulting in a high concentration of landscape ecological risks. Dongxiang County and Linxia County are close to Linxia City, and their economic development is relatively fast. Regional development has shown that the ecological environment restricts economic development [33]. Ecological risks have also been aggravated due to excessive economic development. Finally, Xiahe County, Zhuoni County, and Luqu County are restricted by natural conditions, their economic development is relatively slow, the population is small, and human activities are relatively weak. At the same time, the vegetation in Gannan has generally improved since 2000. The increase in vegetation in Xiahe County, Luqu County and Zhuoni County is the most obvious [34], so the landscape ecological risk is relatively low.

The partition management of EFZUYR is of great significance for improving the ecological environment of the research area and promoting a virtuous cycle of the ecosystem. Conducting partitioning based on the different ecological environments means proposing a more targeted ecological environment management plan and future development plan [35,36], which is a suitable solution. The following suggestions are made for different ecological risk areas: (1) ecological restoration should be emphasized in areas with the highest, higher, and medium risks, such as Linxia City, Linxia County, Hezuo City and others with higher economic development levels. In these areas, the human demand for natural systems is accelerating, leading to imbalances in natural systems and a significant loss of biodiversity [37]. Therefore, these areas should strictly control the construction area and reduce land abuse. By rationally adjusting land use and increasing the green area in urban areas, the ecological benefits of urban areas will be improved, and financial and technical support for environmental protection will be strengthened. In Maqu County, where the natural environment is poor, excessive development must be prohibited to reduce human interference. As the recharge water volume of Maqu County accounts for 45% of the total flow of the Yellow River [38], nine provinces along the middle and lower Yellow River in China will be severely affected if the landscape ecological risks of Maqu County further rise. (2) The lowest and lower risk regions should emphasize protecting the original landscape. For example, areas such as Xiahe County and Zhuoni County should actively respond to national, provincial, and municipal ecological protection policies to continuously improve their ecological environment. Since ethnic clustering regions are endowed with unique cultural, historical, and natural landscape values, ecotourism can be carried out within the carrying capacity of their ecological environments to increase the fiscal revenue, which can be invested t back into the ecological construction projects. (3) The ecological security of the research area is highly dependent on natural climatic conditions. It is not easy to support the continued improvement of the ecology of the whole area with the measures taken by humans such as ecological construction and protection engineering. Therefore, the critical points for the development of EFZUYR are balanced between coordinating the conservation of the ecological environment and pursuing sustainable economic development, formulating corresponding partition control measures, and maximizing the role of the research area as a safety barrier for the entire river basin.

### 4.3. The Advantage and Limitations of This Research

The study explored the spatial and temporal evolution of land use change and landscape ecological risk in EFZUYR, supported by long-term data. The advantage of using long-term data is that they can eliminate the effects of the short-term data mutation on the research results, reducing uncertainty [39]. In addition, the ecological risk evaluation method based on landscape pattern in this study, to a certain extent, gets rid of the traditional ecological risk evaluation inherent model of “risk source identification-receptor analysis-exposure and hazard evaluation” and pays more attention to the spatial and temporal change characteristics of risk, which helps to better elucidate understand the current situation of regional ecological risk [20]. Furthermore, the optimization of landscape spatial pattern is closely related to land planning and design [40], this study fully analyzes the change of land use landscape pattern in EFZUYR, which can effectively provide support for the sustainable development of land use in the area. However, there are still some limitations. For example, the data underlying this study are land use data, and the accuracy of land use data interpretation significantly impacts the study. Errors in the characteristics of remote sensing data and technical methods, etc., may lead to uncertainty in the assessment results. Although the study adopted unified land use classification standards in the data processing to eliminate the errors of remote sensing data as much as possible and ensure the accuracy of the data, data errors still exist. Additionally, the spatial and temporal evolution of landscape ecological risk is an integrated and complex process influenced by various aspects of natural and human activities. Therefore, further research and analysis are needed and, thus, a more accurate assessment of regional landscape ecological risks is needed.

## 5. Conclusions

Using the theoretical knowledge of landscape ecology, this study constructed an ecological risk index based on the landscape ecological index and analyzed the spatial and temporal pattern evolution and spatial clustering of ecological risks in EFZUYR with the help of spatial statistical analysis methods. The main conclusions are:

(1) From 1990 to 2018, land use showed different changes. Here, built-up areas and grassland showed the largest increase and the largest decrease, with an increase of 148.84 km² and a decrease of 130.13 km², respectively. Among different types of land transfer, the transfer among cultivated land, grassland, and woodland had prominent advantages, with a transfer area of 5023.37 km², which accounted for 87.16% of the changing area. The order of change rate was as follows: built-up areas > unused land > cultivated land = water area > woodland = grassland. Among them, the dynamic degree of built-up areas is the only one that always showed a growth trend during the research period.

(2) During the study period, the landscape index changes in various land use types had obvious differences. As for the degree of fragmentation, built-up areas were the highest and decreased year by year, with the value dropping from 0.1053 to 0.07962, and the changes in other land types were relatively small. In terms of separation degree, the value of built-up areas dropped from 1.8213 to 1.2911, which was the most dramatic change. The separation degree of water fluctuated from 0.41 to 0.68 and then fell to 0.51. The value of other land use did not change significantly. Regarding the dominance degree, the values of grassland and woodland were maintained at 0.59 and 0.41, respectively, for a long period of time, occupying an absolute advantage. The dominance degree of other land use was below 0.2. For the interference degree, the value of built-up areas was the largest, and the value showed a downward trend, from 0.666 to 0.4477. The interference degree of water fluctuated, increasing from 0.1351 to 0.2172 and then decreasing to 0.1675. The value of cultivated land and unused land increased slightly. In terms of the loss degree, the value of water was the highest, with a value above 0.67, reaching the highest value of 1.0858 in 2010. The loss degree of built-up areas was above 0.44, in second place. The value of woodland was the smallest, which was maintained at around 0.15 for a long time.

(3) The ecological risks of EFZUYR presented a fluctuant rising, falling and then rising trend. The area of the low-risk regions accounted for more than 70% of the entire area, and the overall ecological risk of the area was relatively low. The ecological risks in the EFZUYR long maintained the spatial distribution characteristic of “high at the north and south ends and low in the middle.” In addition, the spatial clustering characteristics of high-risk regions were more obvious: two large-scale hot-spot regions formed in the north and south, and cold spot regions mainly concentrated in the central area, with a decrease in the clustering degree.

## Figures and Tables

**Table 1:** Dynamics of different land use in EFZUYR.

Type of Land Use	Dynamic Degree of Land Use from 1990 to 2000 (%)	Dynamic Degree of Land Use from 2000 to 2010 (%)	Dynamic Degree of Land Use from 2010 to 2018 (%)	Dynamic Degree of Land Use from 1990 to 2018 (%)
Cultivated Land	0.74	−0.39	−0.18	0.04
Woodland	−0.16	0.05	0.02	−0.02
Grassland	−0.06	0.05	−0.06	−0.02
Water	−0.33	−3.27	6.21	0.04
Built-up areas	1.07	3.53	0.69	1.51
Unused land	0.00	−0.05	−0.29	−0.08

**Table 2:** Land use transfer matrix in ecological function zones of the Upper Yellow River from 1990 to 2018.

Type of Land Use		Area of Land Use in 2018 (km²)					
		Cultivated Land	Woodland	Grassland	Water	Built-Up Areas	Unused Land
Area of land use in 1990 (km²)	Cultivated Land	3064.99	8.72	3291.26	9.36	3162.44	8.99
	Woodland	7675.08	21.82	7553.57	21.48	7594.41	21.60
	Grassland	21,475.31	61.07	21,353.60	60.72	21,455.15	61.01
	Water	321.99	0.92	311.49	0.89	209.52	0.60
	Built-up areas	252.04	0.72	278.95	0.79	377.32	1.07
	Unused land	2377.96	6.76	2378.52	6.76	2367.47	6.73

**Table 3:** Changes in the fragmentation degree (C), the separation degree (N), the dominance degree (K), the interference degree (S), and the loss degree(R).

Type of Land Use	Year	C	N	K	S	R
Cultivated Land	1990	0.0047	0.1163	0.1584	0.0536	0.2142
	2000	0.0048	0.1138	0.1683	0.0539	0.2155
	2010	0.0050	0.1180	0.1655	0.0550	0.2198
	2018	0.0052	0.1206	0.1635	0.0556	0.2224
Woodland	1990	0.0083	0.0975	0.4212	0.0764	0.1527
	2000	0.0085	0.0992	0.4168	0.0765	0.1530
	2010	0.0085	0.0990	0.4189	0.0767	0.1533
	2018	0.0084	0.0985	0.4154	0.0762	0.1523
Grassland	1990	0.0011	0.0217	0.5977	0.0670	0.2009
	2000	0.0012	0.0220	0.5955	0.0668	0.2005
	2010	0.0011	0.0209	0.5935	0.0663	0.1988
	2018	0.0012	0.0219	0.5944	0.0667	0.2001
Water	1990	0.0064	0.4190	0.0560	0.1351	0.6757
	2000	0.0065	0.4289	0.0560	0.1382	0.6909
	2010	0.0113	0.6875	0.0416	0.2172	1.0858
	2018	0.0100	0.5179	0.0616	0.1675	0.8377
Built-up areas	1990	0.1097	1.9559	0.1348	0.6660	0.6660
	2000	0.1053	1.8213	0.1376	0.6233	0.6233
	2010	0.0811	1.3746	0.1460	0.4757	0.4757
	2018	0.0762	1.2911	0.1470	0.4477	0.4477
Unused land	1990	0.0039	0.1199	0.1480	0.0531	0.3186
	2000	0.0039	0.1207	0.1478	0.0534	0.3202
	2010	0.0042	0.1256	0.1556	0.0558	0.3346
	2018	0.0044	0.1287	0.1563	0.0569	0.3412

**Table 4:** The area and proportion of different risk levels from 1990 to 2018.

Risk Level	1990		2000		2010		2018	
	Area (km²)	Proportion (%)	Area (km²)	Proportion (%)	Area (km²)	Proportion (%)	Area (km²)	Proportion (%)
Lowest Risk	16,655.00	47.36%	16,509.25	46.94%	16,440.50	46.75%	16,331.00	46.44%
Lower Risk	10,113.50	28.76%	10,093.25	28.70%	10,660.25	30.31%	9341.75	26.56%
Middle Risk	4627.75	13.16%	4725.00	13.44%	4278.75	12.17%	5167.00	14.69%
Higher Risk	1514.25	4.31%	1571.75	4.47%	1644.75	4.68%	2019.00	5.74%
Highest Risk	2258.25	6.42%	2269.50	6.45%	2144.50	6.10%	2310.00	6.57%

**Table 5:** Related parameters of the General G Index.

Year	Observed General G	Expected General G	z-Score	*p*-Value
1990	0.002394	0.00237	4.742	0.000
2000	0.002396	0.00237	5.123	0.000
2010	0.002455	0.00237	13.328	0.000
2018	0.002428	0.00237	10.156	0.000

## Data Availability

Not applicable.

## Appendix A

**Table A1:** The area and proportion of different land use in EFZUYR from 1990 to 2018.

Type of Land Use	1990		2000		2010		2018	
	Area (km²)	Proportion (%)	Area (km²)	Proportion (%)	Area (km²)	Proportion (%)	Area (km²)	Proportion (%)
Cultivated Land	3064.99	8.72	3291.26	9.36	3162.44	8.99	3112.06	8.87
Woodland	7675.08	21.82	7553.57	21.48	7594.41	21.60	7610.14	21.68
Grassland	21,475.31	61.07	21,353.60	60.72	21,455.15	61.01	21,345.18	60.81
Water	321.99	0.92	311.49	0.89	209.52	0.60	326.56	0.93
Built-up areas	252.04	0.72	278.95	0.79	377.32	1.07	400.88	1.14
Unused land	2377.96	6.76	2378.52	6.76	2367.47	6.73	2305.83	6.57

Equation (A1)

The dynamic degree of land use can demonstrate the number of different land use changes in a certain period. It can be used to quantitatively describe the strength of the variance of different land use in the research area. The specific calculation formula is as follows:

(A1) $$K=\frac{U_{t}-U_{0}}{U_{0}}\times \frac{1}{T}\times 100\%$$

In Equation (A1), *K* represents the dynamic degree of a certain land use in a certain period; *U_t_* and *U*_0_ are the amounts of different land use at the beginning and end of the study period; *T* is the research interval. The absolute value of *K* indicates the transferring speed of a certain land use. When *K* is greater than zero, the area of land use types shows an increasing trend, but if *K* is less than zero, the area of land use types shows a decreasing trend.

Equation (A2)

Landscape ecological risk index is composed of landscape disturbance index and landscape vulnerability index, reflecting the relationship between landscape pattern based on land use and ecological risk. The calculation formulas are as follows:

(A2) $$ERI_{k}=\sum_{i=1}^{m}\frac{A_{ki}R_{i}}{A_{k}}$$

In Equation (A2), *ERI* is the landscape ecological risk index of ecological research unit *k*; *i* represents different land use landscapes; *A_ki_* represents the type *i* landscape area of ecological unit *k*; *A**_k_* is the total area of ecological unit; *R**_i_* is the landscape loss degree index of the type *i* land use, which is calculated from the landscape pattern index and the specific required landscape index is shown in Table A2.

Equations (A3)–(A5)

Global statistics such as high/low clustering (Getis-Ord General G) are used to evaluate the overall pattern and trend of the data. The score of z has a greater impact on the research results. If the value of z score is positive, the observed General G index will be larger than the expected one, indicating that high-value attributes will cluster in the research area. If the value of z score is negative, the observed General G index will be smaller than the expected one, showing that low-value attributes will cluster in the research area.

The local spatial autocorrelation of landscape ecological risk describes the spatial correlation characteristics among the attribute values of ecological risk units with the Getis-Ord Gi* index, characterizing the spatial distribution of cold and hot spots. Hot spots indicate the areas where the high values of landscape ecological risks cluster and cold spots represent the areas where the low values of landscape ecological risks cluster. The calculation formula is as follows:

(A3) $$G_{i}^{*}=\frac{\sum_{j=1}^{n}w_{i,j}x_{j}-\bar{X}\sum_{j=1}^{n}w_{i,j}}{S\sqrt{\frac{\left[n\sum_{j=1}^{n}w_{{}_{i,j}}^{2}-{\left(\sum_{j=1}^{n}w_{i,j}\right)}^{2}\right]}{n-1}}}$$

(A4) $$\bar{X}=\sum_{j=1}^{n}x_{i}/n$$

(A5) $$S=\sqrt{\frac{\sum_{j=1}^{n}x_{i}}{n}-{\left(\bar{X}\right)}^{2}}$$

In Equations (A3)–(A5), *X_i_* and *X_j_* represent the *ERI* of ecological risk cells *i* and *j*, respectively. *W_ij_* is the binary matrix of the adjacent space; when ecological cell *i* is adjacent to ecological cell *j*, *W_ij_* = 1; otherwise, *W_ij_* = 0. $\bar{X}$ and *S* are the mean value and standard deviation.

**Table A2:** The construction method of landscape pattern index.

Landscape Index	Calculation Formula	Ecological Meaning
Landscape Fragmentation Degree Index (*C_i_*)	$C_{i}=n_{i}/A_{i}$	In the formula, *n_i_* is the patch number of the type *i* landscape; *A_i_* is the distribution area of type *i* landscape. This index can reflect the degree of fragmentation of different landscape types and partly reflect the complexity of the spatial distribution of the landscape as well as the interference by human activities in the landscape. The smaller the landscape fragmentation, the higher the continuity of different landscape patch types distribution in the research unit. Additionally, the more significant the proportion of the area of different patch types, the more stable the corresponding landscape ecosystem.
Landscape Separation Degree Index (*N_i_*)	$N_{i}=\frac{1}{2}\sqrt{\frac{n_{i}}{A}}\times A/A_{i}$	In the formula, *A* is the total area of all landscapes. Its value represents the separation degree between patches in the same landscape type. The greater the landscape separation degree, the longer the distance between the patch boundaries of the same landscape type, and the higher the heterogeneity of the landscape patch types in the research unit.
Landscape Dominance Degree Index (*K_i_*)	$K_{i}=\frac{\left(B_{i}+L_{i}\right)}{4}+\frac{D_{i}}{2}$	In the formula, *B_i_* equals the ratio between the quadrat numbers of patch *i* and the total quadrat number; *L_i_* equals to the ratio between the numbers of patch *i* and the total patch number; *D_i_* equals to the ratio between the distribution areas of patch *i* and the total area of the quadrat. Its value can reflect whether the distribution of a certain landscape type occupies the dominant position of the landscape. The landscape type with a greater dominance degree can directly affect the evolution of the landscape pattern.
Landscape Interference Degree Index (*S_i_*)	$S_{i}=aC_{i}+bN_{i}+cK_{i}$	In the formula, a, b, c are the weights of their corresponding landscape indexes, and the sum of a, b and c equals one. Based on the analysis and combined with the research experience, a, b and c are assigned with the weights of 0.6, 0.3, and 0.1. These values are used to express the interference degree of human production activities on different types of landscapes.
Landscape Vulnerability Degree Index (*F_i_*)	Obtained by expert scoring, assignment and normalization	It refers to the vulnerability of the landscape ecosystem when encountering different factors. This value has a greater relationship with the level of the landscape ecosystem. Generally, the lower the ecosystem level, the higher the internal vulnerability of the system. According to the research results, the vulnerability of unused land, water, cultivated land, grassland, woodland, and built-up areas is 6, 5, 4, 3, 2 and 1.
Landscape Loss Degree Index (*R_i_)*	$R_{i}=S_{i}\times F_{i}$	The loss degree is related to each stage in the development of the landscape ecosystem. Typically, the lower the level of the landscape ecosystem, the higher its vulnerability. Conversely, the higher the level of the landscape ecosystem, the more stable its internal organizational structure and the lower the interference degree, and therefore the lower the vulnerability.
